# The economic burden of measles in children under five in Uganda

**DOI:** 10.1016/j.jvacx.2020.100077

**Published:** 2020-09-09

**Authors:** Gatien De Broucker, Anthony Ssebagereka, Rebecca Racheal Apolot, Mutebi Aloysius, Elizabeth Ekirapa Kiracho, Bryan Patenaude, Dagna Constenla

**Affiliations:** a International Vaccine Access Center, Johns Hopkins Bloomberg School of Public Health, United States; b Makerere University School of Public Health, Uganda; c GlaxoSmithKline Plc., Panama City, Panama

**Keywords:** Measles, Cost-of-illness, Outbreak, Uganda, Vaccine-preventable disease, Economic burden, Economic benefit of immunization

## Abstract

**Background:**

There is very limited evidence about the economic cost of measles in low-income countries. We estimated the cost of treating measles in Uganda from a societal perspective.

**Methods:**

We conducted an incidence-based cost-of-illness study in Uganda. We surveyed the facility staff, recording hospital-related expenditures for measles patients. We interviewed caregivers of children with measles at 48 selected healthcare facilities. We conducted phone interviews with caregivers 7–14 days post-discharge to capture additional out-of-pocket expenses and time costs.

**Results:**

From a societal perspective, a hospitalized and an ambulatory episode of measles cost 2018 US$ 60 and $15, respectively. The government spent on average $12 and $5 per hospitalized and ambulatory episode of measles. Including both public and private facilities, caregivers incurred approximately $44 in economic costs, including $23 in out-of-pocket expenses. In 2018, 2614 cases of measles were confirmed, resulting in $135,627 in societal costs, including $59,357 in economic costs to Ugandan households.

**Conclusion:**

This cost-of-illness study is the first to use empirical methods to quantify the economic burden of measles in a low-income country. Information related to the cost of treating measles is important for guiding decisions related to changes in measles control and prevention.

## Highlights

• Measles costed over $135,627 in societal costs for 2018, incl. $59,357 to households.
• Ugandan caregivers faced $44 in economic costs incl. $23 in out-of-pocket payments.
• Measles deepens the inequalities in access to healthcare in the population.

## 1 Background

The Government of Uganda subsidizes one dose of measles-containing vaccine (MCV1) in their expanded program for immunization (EPI), delivering the vaccine through the routine immunization program and national measles campaigns. WHO/UNICEF Estimates of National Immunization Coverage (WUENIC) for MCV1 grew from 59% in early 2000 to 79% in 2017 and 86% in 2018 [1]. In addition to strengthening routine immunization, supplemental immunization activities (SIA) were conducted in 2003 and 2006 as part of the 2002–2006 measles control strategy implemented in Uganda [2], [3]. These recent numbers are consistent with the global estimate of 86% for MCV1 and 69% for the second dose (MCV2). The number of measles cases in children dropped to 10 cases in 2004 following the 2003 SIA.

While MCV2 could not be added to the EPI, the Ministry of Health conducted several SIA, with the latest in October 2015 [4]. Between 2016 and 2018, the government could not fund SIA to help achieve the 90% measles immunization coverage required to effectively stop measles transmission [5]. With a significant number of children unimmunized, measles cases spiked in 2018 from 139 confirmed cases in 2016 to 1,021 in 2017 and 2,627 in 2018 [6].

In the context of a resurgence of measles globally, the government of Uganda made measles control a priority in 2019, evaluating different strategies to curb its incidence [5]. Estimates of the economic burden that both the government and households bear can help make informed decisions regarding changes to measles control and prevention in Uganda. To our knowledge, this is the first cost-of-illness (COI) study producing real-world cost estimates for measles in a low-income country (LIC) [7]. This study is part of a larger stand-alone cost-of-illness study generating estimates of the cost of measles, pneumonia and diarrhea for the healthcare facility, caregiver and society in Uganda.

## 2 Methods

### 2.1 Study design

We conducted an incidence-based study with an ingredient-based approach to estimate the cost of treatment and productivity loss of an acute episode of measles from the societal and household perspectives. The costs of measles-related sequelae beyond 14 days post the initial interview at discharge were not considered in the study. The Uganda National Council for Science and Technology (IRB: HS 2131), Makerere University School of Public Health (IRB: Protocol 439) and the Johns Hopkins Bloomberg School of Public Health (IRB #7256) examined the risks and benefits related to this research project and granted ethical approval. The data are available in open access [21].

### 2.2 Study population and sites

**Fig. 1:**
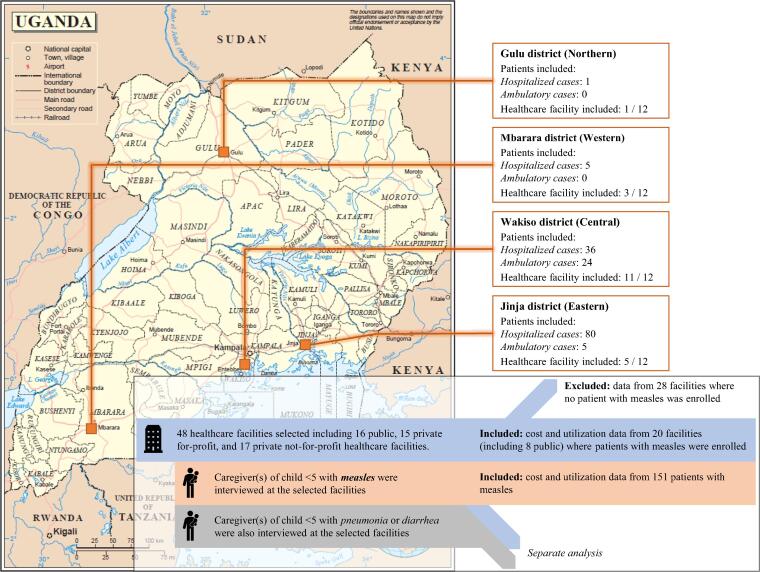
Map of the study sites, based on the United Nations Map No. 3862 Rev.4, May 2003.

The study took place in four districts representing all regions of Uganda: Gulu district (Northern Region), Jinja (Eastern), Mbarara (Western) and Wakiso (Central). Healthcare facilities in urban and rural areas were selected. We selected a total of 48 healthcare facilities: 16 public, 15 private for-profit and 17 private not-for-profit facilities (see Supplementary methods for further details). Twenty of the 48 facilities reported measles cases (including eight public facilities) and were included in the analysis. Based on the recommendations of the healthcare facilities’ staff, we selected a total 282 pharmacies from the area surrounding the facilities. Pharmacies were all privately owned and registered (see Fig. 1).

We recruited the adult caregiver of children (0–59 months) suspected of having measles to understand their perspective. Cases were selected based on the discharge diagnosis, which was clinically confirmed based on the WHO clinical case definition [8]. Suspected measles cases with comorbidities were excluded.

### 2.3 Data collection

From August 2017 to July 2018, we interviewed administrators and managers of the 48 selected healthcare facilities and the district health offices they represented, as well as medical staff, laboratory technicians, statisticians and storekeepers, to obtain resource utilization and expenditure data from the healthcare provider’s perspective. Whenever possible, we used administrative data and reports to adjust the reported estimates. Healthcare facility costs included operating costs [9]: labor and overhead costs, and itemized costs for medical supplies and medications used for diagnostic tests, hospitalization and treatment. We considered as capital costs the medical equipment used to care for measles cases (*e.g.*, microscopes, laboratory instruments). For tertiary- and secondary-level hospitals, data collection was restricted to the pediatric ward.

There were two healthcare facility surveys administered. The first was a one-time healthcare facility survey that collected the capital and operating costs, and the average time spent by the healthcare professionals (*e.g.*, physicians, registered nurses, enrolled nurses, etc. – excluding administrative staff) treating measles cases. The second was a monthly healthcare survey that collected the number of cases and whether facilities experienced medications or supplies stock-outs. We also administered a one-time survey for the four district health offices to collect additional data on medication costs in the public sector as this was unavailable in healthcare facilities. A one-time pharmacy survey was performed to estimate medication costs from the private sector perspective.

Caregivers were interviewed at the time of discharge from the facility in person and 7–14 days later by phone, capturing all costs incurred at the facility where they received treatment, in previous facilities, and after discharge. We obtained information about the caregivers’ out-of-pocket payments related to the measles episode, including information about direct medical costs (registration fees, medications, medical procedures, hospitalization) and non-medical costs (transportation to and from the facilities and meals). To estimate the indirect cost, we also asked caregivers information about the time spent providing care for the child at the facility and traveling to and from the facilities. Additionally, we collected information about their household, their daily expenditures and their income to assess their socio-economic status.

### 2.4 Costing methods

All costs were patient-specific apart from overhead, labor and capital costs. The latter costs were shared with all other patients in the pediatric ward. Capital costs were annualized based on a standard lifetime of 5 years for medical equipment with a discount rate of 3%. Measles treatment could not be associated with a specific area of each facility and specific personnel time as recommended by the Global Health Cost Consortium (GHCC) to calculate operating costs [9]. The sum of the annualized overhead, labor and capital costs attributable to an episode of measles was calculated using patient-days (Eq. (1)).

(1) $$S=\overset{n\;;\;m}{\underset{i=0\;;\;j=0}{\sum }}\frac{c_{j}\times {\mathrm{los}}_{i,j}}{p_{j}}$$

where *S* is the total cost of overhead, labor and capital attributable to measles, *c_j_* the total annual cost, *p_j_* the annual number of patients who used the facility and *los_i__,j_* the length of stay in days for caregiver *i* over *n* total caregivers, and for facility *j* over *m* total healthcare facilities.

For the few facilities with a ward dedicated to measles treatment, the capital costs associated to this ward were calculated based on the utilization rate of the measles ward only, while the capital costs shared across the pediatric ward used the utilization rate of the whole pediatric ward. Data on the cost and the lifetime of medical equipment, furniture and infrastructure were either not known or not deemed reliable by respondents at the healthcare facilities. Item costs were drawn from supplemental data collection at the district health offices and their lifetime was assumed for the calculation of capital costs. All other costs were combined with patient-specific utilization.

For caregivers, all direct costs were itemized. Indirect costs were estimated through a human capital approach, combining the head of the household’s average income and the time spent getting to/from and in the healthcare system. Based on stakeholders’ feedback, we also reported detailed time loss exempt of monetary valuation.

We examined whether there were any differences in direct, indirect and overall costs for an episode of measles based on the child’s or the caregiver’s gender, the household’s residence, type of visit and facility, and length of stay using an independent *t*-test. For age groups, study areas and asset quintiles, we performed a one-way ANOVA. We used the Wilcoxon and Kruskal-Wallis rank tests when either the hypothesis of normal distribution or equal variance was rejected.

The societal costs are the combination of the costs borne by the caregivers and the healthcare system. We assumed that all costs borne by private healthcare were transferred to caregivers through charges and copayments. We estimated the country-level costs for measles by combining the costs per episode with the national incidence rate estimates.

Costs are reported in 2018 US dollars ($). We used the conversion rate of $1 = UGX 3727 for 2018 [10]. Cost categories were defined based on the GHCC and Jo [9], [11].

### 2.5 Principal component analysis

The socioeconomic status of each household was defined based on asset scores generated through a principle component analysis (PCA) approach. The PCA considered the ownership of durable assets in the households [12]: the households’ dwelling characteristics (*e.g.*, wall, roof and floor materials, water and sanitation facilities, and utilities) and durable goods (*e.g.*, radio and television). Based on their asset score ranking, the households were divided into asset quintiles.

### 2.6 Catastrophic health expenditures

Catastrophic health expenditures were calculated using the share of direct cost (medical and non-medical combined) over the monthly income and expenditures of the household. Monthly expenditures comprised of food, clothing, supplies, leisure, tax paid, other healthcare expenses and other expenses. A household experienced catastrophic health expenditures when it spent more than the following thresholds on this episode of measles: 10% of its income, 25% of its monthly expenditures or 40% of its monthly expenditures without food [13].

## 3 Results

We captured a total of 151 measles episodes during the data collection period. Most measles cases came from outbreaks in 2 districts, with 60 affected children (40%) in Wakiso district and 85 (56%) in Jinja district. Children with measles were found mainly in public healthcare facilities (101 children, 67%), followed by private not-for-profit facilities (39, 26%) and private for-profit facilities (11, 7%). Most cases were hospitalized (122, 81%). Most children were over the age of 1 year (96 children, 63%) with equal proportion of males and females. Over 94% of the caregivers were women. Most caregivers had completed primary school (104, 69%), were living in rural areas (74, 49%) and came from larger households, 70 (46%) from households with 4–5 people and 47 (31%) with 6 people or more.

Almost all caregivers reported that the child had been vaccinated (any vaccine) and had an immunization card (96%), although only 22 (15%) caregivers had the card at the time of the interview. In our sample, 10 children (45%) who presented their immunization cards had MCV1 recorded, 2 (9%) had MCV2 recorded, and 12 (55%) did not have any measles vaccine recorded.

### 3.1 Cost-of-illness estimates

The mean economic cost per episode of measles for caregivers was UGX 109,461 with an average out-of-pocket cost of UGX 59,009, corresponding respectively to 2018 US$ 29 and $16. Table 1 illustrates the caregiver perspective in detail.

**Table 1:** Total household costs for an episode of measles (costs in 2018 US dollars and time loss in days or hours).

							INPATIENT CARE									
Timing	Type of cost	Public (n = 84)					Private for-profit (n = 6)					Private not-for-profit (n = 32)				
		Mean	SE	95% CI		n(c > 0)	Mean	SE	95% CI		n(c > 0)	Mean	SE	95% CI		n(c > 0)
Before current visit^A^	Direct medical	$1.22	$0.57	$0.09	$2.36	14	$0.00	$0.00	$0.00	$0.00	0	$0.52	$0.24	$0.04	$1.01	6
	Direct non-medical	$0.66	$0.19	$0.29	$1.04	21	$2.74	$1.48	-$1.06	$6.53	3	$0.84	$0.40	$0.02	$1.66	7
	Indirect	$0.99	$0.32	$0.35	$1.63	30	$1.56	$0.93	-$1.01	$4.13	3	$3.01	$1.59	-$0.24	$6.26	9
	*Time loss [days]*	*0.34*	*0.10*	*0.14*	*0.54*	31	*0.33*	*0.19*	*−0.15*	*0.80*	4	*0.45*	*0.16*	*0.12*	*0.79*	9

Current visit	Direct medical	$3.81	$0.50	$2.81	$4.82	57	$12.74	$8.50	-$9.12	$34.61	4	$13.45	$2.31	$8.73	$18.17	26
	Direct non-medical	$7.92	$0.57	$6.79	$9.05	84	$16.68	$4.90	$4.08	$29.28	6	$7.19	$1.30	$4.53	$9.84	31
	Indirect	$14.14	$2.37	$9.42	$18.85	80	$33.76	$11.83	$0.93	$66.60	5	$17.78	$4.76	$8.06	$27.51	30
	*Time loss [days]*	*3.30*	*0.22*	*2.87*	*3.74*	83	*3.27*	*0.31*	*2.47*	*4.07*	6	*3.98*	*0.54*	*2.88*	*5.08*	32

Follow-up^A^	Direct medical	$2.25	$0.48	$1.30	$3.20	28	$2.46	$2.46	-$3.86	$8.78	1	$9.34	$1.25	$6.77	$11.91	18
	Direct non-medical	$7.15	$0.86	$5.43	$8.87	48	$3.17	$3.17	-$4.99	$11.34	1	$5.58	$0.84	$3.85	$7.31	21
	Indirect	$9.31	$1.82	$5.67	$12.95	38	$30.68	$30.68	-$54.50	$115.85	1	$13.99	$2.68	$8.46	$19.52	20
	*Time loss [days]*	*2.94*	*0.42*	*2.10*	*3.77*	39	*0.68*	*0.68*	*−1.07*	*2.43*	1	*5.07*	*0.67*	*3.69*	*6.46*	21

**Total out-of-pocket expenses**		**$21.79**	**$1.45**	**$18.91**	**$24.67**	**84**	**$37.80**	**$8.46**	**$16.04**	**$59.55**	**6**	**$34.13**	**$2.92**	**$28.17**	**$40.08**	**32**
**Total economic cost**		**$44.25**	**$3.65**	**$36.98**	**$51.51**	**84**	**$92.80**	**$42.23**	**-$15.77**	**$201.36**	**6**	**$65.20**	**$5.74**	**$53.49**	**$76.90**	**32**

							OUTPATIENT CARE									
Timing	Type of cost	Public (n = 17)					Private for-profit (n = 5)					Private not-for-profit (n = 7)				

Mean	SE	95% CI		n(c > 0)	Mean	SE	95% CI		n(c > 0)	Mean	SE	95% CI		n(c > 0)

Before current visit^A^	Direct medical	$0.00	$0.00	$0.00	$0.00	0	$0.38	$0.38	-$0.67	$1.42	1	$1.72	$1.72	-$2.50	$5.95	1
	Direct non-medical	$0.00	$0.00	$0.00	$0.00	0	$1.29	$1.29	-$2.29	$4.86	1	$0.00	$0.00	$0.00	$0.00	0
	Indirect	$0.00	$0.00	-$0.01	$0.01	1	$0.05	$0.05	-$0.11	$0.21	1	$0.03	$0.03	-$0.05	$0.11	1
	*Time loss [hours]*	*0.01*	*0.01*	*−0.02*	*0.05*	1	*0.47*	*0.39*	*−0.61*	*1.55*	2	*0.05*	*0.05*	*−0.07*	*0.16*	1

Current visit	Direct medical	$0.29	$0.18	-$0.09	$0.67	3	$16.53	$3.37	$7.17	$25.88	5	$2.40	$1.67	-$1.69	$6.49	2
	Direct non-medical	$1.74	$0.67	$0.33	$3.16	14	$1.88	$0.49	$0.52	$3.23	5	$0.35	$0.27	-$0.31	$1.00	2
	Indirect	$1.90	$0.32	$1.20	$2.59	14	$2.25	$0.21	$1.58	$2.93	4	$3.95	$1.72	-$0.27	$8.16	7
	*Time loss [hours]*	*2.77*	*0.31*	*2.13*	*3.42*	17	*3.00*	*0.53*	*1.54*	*4.46*	5	*3.62*	*0.62*	*2.11*	*5.13*	7

Follow-up^A^	Direct medical	$0.47	$0.47	-$0.53	$1.48	1	$16.88	$4.80	$3.54	$30.22	4	$0.00	$0.00	$0.00	$0.00	0
	Direct non-medical	$0.03	$0.03	-$0.04	$0.10	1	$1.82	$0.60	$0.17	$3.48	4	$0.00	$0.00	$0.00	$0.00	0
	Indirect	$0.03	$0.03	-$0.03	$0.09	1	$2.41	$0.34	$1.34	$3.49	4	$0.00	$0.00	$0.00	$0.00	0
	*Time loss [hours]*	*0.13*	*0.13*	*−0.14*	*0.40*	1	*2.70*	*0.87*	*0.29*	*5.11*	4	*0.00*	*0.00*	*0.00*	*0.00*	0

**Total out-of-pocket expenses**		**$2.54**	**$0.79**	**$0.87**	**$4.21**	**15**	**$38.78**	**$7.36**	**$18.34**	**$59.21**	**5**	**$4.47**	**$2.94**	**-$2.72**	**$11.66**	**2**
**Total economic cost**		**$4.13**	**$0.97**	**$2.06**	**$6.20**	**17**	**$42.55**	**$8.20**	**$19.78**	**$65.32**	**5**	**$8.45**	**$3.16**	**$0.72**	**$16.18**	**7**

*Notes:* SE, Standard Error; n, number of caregivers; n(c > 0), number of caregivers who incurred costs greater than $0.

A Includes costs incurred at public and private healthcare facilities and providers.

Most inpatient visits take place in public healthcare facilities (69%), followed by private not-for-profit (26%) and for-profit (5%) facilities. The trend is similar for outpatient visits with most visits happening in public facilities (59%) followed by private not-for-profit (24%) and for-profit facilities (17%). Caregivers in public and private not-for-profit healthcare facilities incurred lower costs than in private for-profit facilities for both types of care. On average for a hospitalized case of measles in a public facility in Uganda, caregivers spent a total of $44, including $22 in out-of-pocket expenses. Caregivers using private not-for-profit facilities faced $65 in economic costs with $34 in out-of-pocket expenses. In contrast, caregivers using private for-profit facilities for hospitalization incurred an average of $93 in economic costs, including $38 in out-of-pocket expenses. Out-of-pocket expenses were similar for caregivers using private for-profit and not-for-profit facilities, with non-medical costs as the main cost to use private for-profit facilities (60% of out-of-pocket expenses) and medical costs for private not-for-profit facilities (63%). Longer lengths of stay (3 days or more) meant significantly higher direct costs ($ 28 compared with $ 21, p = 0.019). There was no significant difference in indirect costs (Table 2).

**Table 2:** Differences in household costs across caregiver characteristics.

Characteristic	n	Direct costs			Indirect costs			Total costs		
		Mean	SE	p-value^B^	Mean	SE	p-value^B^	Mean	SE	p-value^B^
*Age group*										
< 6 months	13	$15.43	$4.50	0.015	$11.04	$3.64	0.195^C^	$26.46	$7.26	0.019^C^
6–11 months	42	$29.88	$2.93		$31.29	$7.29		$61.17	$8.75	
12–24 months	49	$20.44	$2.29		$18.53	$3.08		$38.97	$4.29	
> 24 months	47	$20.59	$2.36		$22.98	$4.61		$43.57	$5.15	

*Gender (child)*										
Female	70	$22.57	$2.21	0.454	$21.03	$3.31	0.617^C^	$43.59	$4.01	0.731^C^
Male	77	$23.45	$1.93		$25.38	$4.55		$48.83	$5.66	

*Gender (caregiver)*										
Female	142	$22.30	$1.43	0.256	$23.67	$2.95	0.835^C^	$45.97	$3.61	0.817
Male	9	$32.26	$8.29		$14.03	$3.24		$46.29	$9.10	

*Study area*										
Gulu	1	$8.85	.	0.000^C^	$121.30	.	0.000^C^	$130.15	#VALUE!	0.000^C^
Jinja	85	$26.37	$1.60		$26.66	$3.82		$53.03	$4.61	
Mbarara	5	$24.44	$14.61		$30.24	$26.18		$54.68	$30.43	
Wakiso	60	$17.64	$2.43		$15.29	$3.31		$32.93	$4.46	

*Residence*										
Rural	74	$26.02	$2.03	0.080	$22.52	$3.65	0.904	$48.53	$4.52	0.828
Semi-urban	18	$22.68	$5.05		$26.76	$10.55		$49.44	$11.31	
Urban	59	$19.27	$2.11		$22.91	$4.58		$42.18	$5.82	

*Type of visit*										
Inpatient	122	$25.60	$1.47	0.000	$27.44	$3.25	0.000^C^	$53.04	$3.81	0.000^C^
Outpatient	29	$10.05	$3.34		$2.95	$0.55		$13.00	$3.53	

*Sector*										
Public	101	$18.66	$1.43	0.000	$20.14	$2.81	0.373^C^	$38.80	$3.53	0.002^C^
Private for-profit	11	$42.86	$5.43		$38.76	$24.41		$81.63	$27.62	
Private not-for-profit	39	$28.62	$3.14		$26.90	$5.29		$55.52	$6.03	

*Length of stay (IPD)*^A^										
< 3 days	103	$21.46	$1.95	0.019^C^	$22.28	$5.38	0.207	$43.74	$6.68	0.044^C^
>= 3 days	18	$28.19	$2.00		$30.86	$4.11		$59.05	$4.54	

*Asset quintiles*										
Poorest	31	$29.83	$2.88	0.028	$20.20	$4.53	0.958^C^	$50.04	$5.29	0.309^C^
2nd	30	$18.75	$3.27		$27.88	$7.71		$46.63	$9.38	
3rd	30	$16.86	$2.37		$22.91	$5.95		$39.77	$6.68	
4th	30	$22.78	$2.88		$18.98	$2.90		$41.76	$5.05	
Richest	30	$25.32	$3.96		$25.84	$8.75		$51.16	$10.95	

*Notes:* SE, Standard Error; n, number of caregivers.

A Length of stay includes only hospitalized cases of measles (n = 121 + 1 with missing length of stay).

B Based on one-way ANOVA (age group, study area and asset quintile) and independent *t*-test for the others.

C Based on Kruskal-Wallis rank test.

For outpatient cases, caregivers using public facilities spent an average of $4, including $3 in out-of-pocket expenses. Caregivers using private not-for-profit facilities spent $8 in total costs with $4 in out-of-pocket expenses. In private for-profit facilities, total costs increased to $43 with $39 in out-of-pocket expenses.

Over the continuum of care for an episode of measles that required hospitalization, the cost of the current visit including indirect costs contributed to 54–61% of the total cost for the episode. For outpatient episodes, this cost was the key driver for public and private not-for-profit facilities with 88% and 79% respectively. In comparison, the costs of the current visit in private for-profit facilities (48%) and of follow-up visits (49%) were similar for outpatient care. Relative to total expenses, indirect costs due to productivity loss were the most important contributor to total expenses, ranging from 43% to 62%, with exception for outpatient care in private for-profit and not-for-profit facilities where medical costs were the key driver, contributing to 78% and 49% of the total cost respectively (Table 1).

Across all age groups, children between 6 and 12 months had the highest rate of hospitalization (89%) and use of private for-profit healthcare facilities (15%), resulting in the highest direct and total costs with $29 and $61 (p < 0.05).

Jinja district had a much higher rate of hospitalization (94%) of measles cases than Wakiso district (60%), both mainly in public facilities (65% and 72% of all cases respectively). This explains the significant difference in direct costs with $26 for Jinja compared to $18 for Wakiso (p < 0.001).

Hospitalization rates decreased with wealthier quintiles from 97% in the 1st quintile to 66% in the 5th, while the use of private for-profit healthcare facilities increased from 0% (1st) to 20% (5th). The use of private not-for-profit facilities was much greater in the 1st quintile (45%) and similar across the other 4 quintiles (16–24%). There was limited significance in the differences in direct, indirect and total costs for both inpatient and outpatient cases across asset quintiles. Interestingly, while the median indirect costs were not different across quintiles, both the income and the time loss were different. Median income was significantly higher in the 3rd, 4th and 5th quintile compared to the poorest 1st quintile, and the median time loss of 9.15 days spent at healthcare facilities for the 1st quintile, significantly higher than 4.68, 4.13 and 3.52 days for the other three quintiles for a hospitalized episode, respectively (p < 0.01).

On average, the government spent $5 per outpatient case and $12 per hospitalized case of measles in Uganda (see Table 3). The cost of treating measles differed by level of facility and by type of care. In Health Centres (HC) II and III, the total cost of care for outpatient care averaged $0.57 and $4.17 respectively. HC IV had an average total cost of care of $7.73 to treat an outpatient case and $16.85 for a hospitalized case of measles. For regional referral hospitals, average total costs were $1.11 for an outpatient case and $12.00 for a hospitalized case.

**Table 3:** Government costs for an episode of measles in 2018 US dollars.

Cost	Inpatient case				Outpatient case			
	*Public Healthcare Centre II*							
	Mean	SE	95% CI		Mean	SE	95% CI	
	Inpatient care not offered				n = 1; hc = 1			
Capital	.	.	.	.	$0.00	.	.	.
Overhead	.	.	.	.	$0.02	.	.	.
Labor	.	.	.	.	$0.32	.	.	.
Medications	.	.	.	.	$0.23	.	.	.
**Total cost**	**.**	**.**	**.**	**.**	**$0.57**	**.**	**.**	**.**

	*Public Healthcare Centre III*							
	Inpatient care not offered				n = 8; hc = 1			
Capital	.	.	.	.	$0.00	.	.	.
Overhead	.	.	.	.	$0.11	.	.	.
Labor	.	.	.	.	$3.68	.	.	.
Medications	.	.	.	.	$0.39	.	.	.
**Total cost**	**.**	**.**	**.**	**.**	**$4.17**	**.**	**.**	**.**

	*Public Healthcare Centre IV*							
	n = 4; hc = 3				n = 7; hc = 2			
Capital	$0.06	$0.03	-$0.05	$0.16	$0.04	$0.01	$0.02	$0.06
Overhead	$0.29	$0.04	$0.17	$0.42	$0.10	$0.01	$0.07	$0.14
Labor	$16.38	$2.58	$8.17	$24.59	$6.82	$0.40	$5.84	$7.81
Medications	$0.12	$0.02	$0.06	$0.19	$0.76	$0.60	-$0.70	$2.22
**Total cost**	**$16.85**	**$2.59**	**$8.61**	**$25.09**	**$7.73**	**$0.20**	**$7.23**	**$8.23**

	*Public Regional Referral Hospital*							
	n = 80; hc = 3				n = 1; hc = 1			
Capital	$0.10	$0.01	$0.08	$0.12	$0.00	.	.	.
Overhead	$2.48	$0.07	$2.34	$2.62	$0.55	.	.	.
Labor	$7.76	$0.45	$6.86	$8.66	$0.55	.	.	.
Medications	$1.67	$0.05	$1.56	$1.77	$0.02	.	.	.
**Total cost**	**$12.00**	**$0.46**	**$11.08**	**$12.92**	**$1.11**	**.**	**.**	**.**

*Note:* n, number of caregivers interviewed at the facility level.

hc, number of healthcare facilities included at the facility level.

The main driver of the facility costs was the personnel cost ranging from 56% (HC II) to 88% (HC III and IV) of the total cost for an outpatient case and 65% (regional referral hospital) to 97% (HC IV) for an inpatient case. Medication costs were also significant at 2–40% for outpatient care and 1–14% for inpatient care. Medication costs are generally distributed between $0.02 (referral hospital) and $0.76 (HC IV) for outpatient cases. For hospitalized cases, they averaged between $0.12 (HC IV) and $1.67 (referral hospital).

### 3.2 Economic burden

**Fig. 2:**
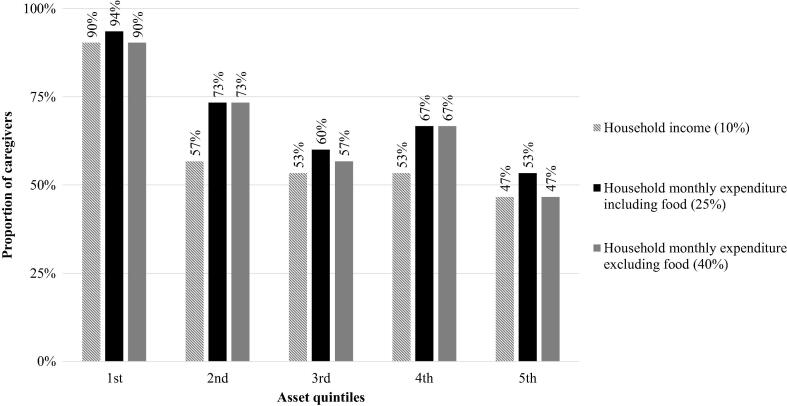
Catastrophic health expenditures related to the episode of measles by asset quintile. Notes: Caregivers were sorted in asset quintiles groups of equal sizes, based on the principal component analysis.

Over 60% of the caregivers reported spending over 10% of the household’s monthly income including all wage-earners in the household on this episode of measles (Fig. 2). Caregivers spent on average 30% of their household income. The economic cost of an episode of measles took over 18% of the annual national gross domestic product (GDP) per capita.

As a share of their household expenditures including food, 70% of caregivers reported spending over 25%. When excluding food, 67% reported spending over 40%. The proportion of households experiencing catastrophic health expenditures decreased with richer quintiles. Over 90% of caregivers’ daily consumption (excluding food) in the poorest 1st quintile faced catastrophic health expenditures, compared to 57%, 67% and 47% in the 3rd, 4th and 5th quintiles, respectively.

Among the richer quintiles, the use of savings increased, while borrowing from family and friends, and taking out a loan decreased. In the poorest (1st) quintile, 20% reported using their savings to pay for inpatient care compared to 70% of the caregivers in the richest (5th) quintile. For outpatient care, the use of savings did not increase with richer asset quintiles. A significant number of caregivers used other sources of funding to pay for their healthcare, from fee waivers at the healthcare facility to other community programs (see Supplementary Material: Fig. S1).

### 3.3 Societal costs and country cost of measles

When weighted to represent the sample utilization rates of each facility (see Supplementary Material), the average societal cost per measles episode across all sectors and types of visits was $52. Hospitalized episodes accounted for an average of $60 per episode, while episodes only requiring ambulatory care had an average of $15.

**Fig. 3:**
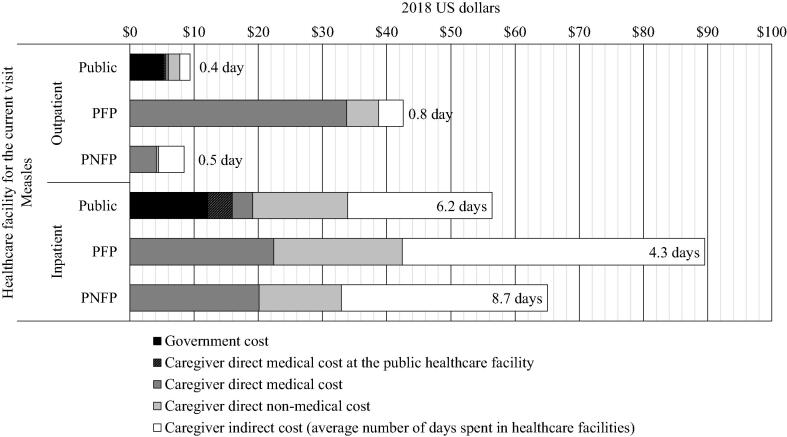
Societal cost of an episode of measles. Notes: PFP, Private for-profit; PNFP, Private not-for-profit. Number of days is the average duration of the cumulative stay in all healthcare facilities visited.

Using public healthcare facilities was associated with the lowest societal cost for inpatient care ($79). Private not-for-profit facilities offered the lowest costs for outpatient care with $35 and second lowest with $87 for inpatient care (Fig. 3). Finally, the use of private for-profit facilities was associated with the highest cost with $55 per outpatient episode and $153 for an inpatient episode. The cost of hospitalized cases in for-profit facilities was driven by indirect costs: at equal time spent on care, the indirect cost for their users was twice as much as for users of private not-for-profit facilities. Medication stock-outs shifted some of the cost expected to be covered by the government to the caregivers.

By the end of 2018 when data collection was completed, there were 2,614 confirmed cases of measles costing over $135,627 in societal cost, including $59,357 that Ugandan households bear in out-of-pocket expenses.

## 4 Discussion

The Ugandan government is committed to promoting universal health coverage throughout the country. However, if the population is to receive these services without financial hardship, attention must be paid to shielding the population from meeting the costs of care. If the government relies on the private sector, whether for- or not-for-profit, most of the medical costs will still be borne by households. Healthcare provided by private for-profit facilities was costlier compared to public and private not-for-profit facilities for each child surviving the disease, particularly for outpatient care. Not only were the medical costs substantially higher, caregivers also had to spend as much in follow-up care: 49% and 35% of their total cost for outpatient and inpatient episodes respectively. The study shows that most of the medical costs was transferred from the caregivers to the government: for inpatient cases, public facilities covered $12 leaving on average $7 for the caregivers to pay, and for outpatient cases, they covered $5 and left $1 for caregivers: mainly medications that were not available at the facility due to stock-outs.

However, increasing and sustaining the required 90% coverage is not enough: immunized children remain at risk during outbreaks. Our sample showed that a significant proportion (45%) of children diagnosed with measles received at least one dose of MCV, although most of them received MCV1 much later than the recommended age of 9 months old [14]: with a median age of 20 months old, ranging from 9 to 58 months. The lack of timeliness for MCV has been previously documented in Uganda and is considered as a risk factor for measles outbreaks [15]. The latest WHO surveillance data showed that a significant number of children diagnosed with measles had been vaccinated [6], potentially indicating that it may not perform as it should in Uganda. Recognized shortcomings in the vaccine effectiveness in Uganda include settings where the cold chain does not perform adequately [16] and where there is a high prevalence of malaria and HIV [17]. Additionally, behavioral factors can bolster the spread of measles: crowded water points [18] and healthcare facilities without effective triage [19] contributed to two separate outbreaks in 2015 and 2016 in Western and Eastern Uganda.

These cost estimates provide invaluable insights to understand the burden measles poses to the healthcare system and to Ugandan households, both in financial and in economic terms. However, they are likely to be conservative as they do not include the long-term costs of measles, including the costs of measles-related sequelae [20].

The study is subject to a number of limitations. While the centralized laboratory at Entebbe Hospital confirmed measles cases, most medical records weren’t updated, relying primarily on the clinical diagnosis of physicians to identify measles cases. Furthermore, only caregivers of surviving children discharged from a healthcare facility were interviewed. We did not record any cost associated with death, or costs related to children that had not been admitted to a healthcare facility.

Significant discrepancies were found between the number of measles cases reported by the healthcare facilities and the number reported in the Health Management Information System. Addressing those shortcomings with local stakeholders, the exclusion of cases with comorbidities (*e.g.*, HIV) from the study and the potential financial or political benefits that may accrue by containing the incidence of measles were brought as possible explanations.

Finally, data on funding were collected for all facilities and included funding from government subsidies and international non-governmental organizations. Such funding may have contributed to covering the cost of treatment of an episode of measles, particularly in private not-for-profit facilities. However, how this funding was processed and how it could be traced down to healthcare activities relevant to measles was not clear and we could not integrate it in our estimates.

## 5 Conclusion

The economic burden of measles in Uganda is substantial, costing $60 (UGX 225,467) per episode in inpatient care, and $15 (UGX 55,430) in outpatient care. The poorest households suffer most from the economic burden of measles outbreaks. Findings of the current study can assist in the allocation of resources for measles treatment, provide inputs for future health economic studies that evaluate the economic value of vaccination programs, as well as serve as the basis for policy and planning relative to control and prevention measles strategies.

## Meetings where this information has previously been presented

Preliminary findings of this research were presented to the Ministry of Health of Uganda on December 4, 2018 in Kampala, Uganda. Regional-specific preliminary findings were presented at the district health office of each district: Jinja (Dec. 5), Gulu (Dec. 7), and Mbarara (Dec. 11). Results for Wakiso district were presented on Dec. 4. Local stakeholders involved in the project, healthcare facility managers, and representatives of international organizations attended these meetings. Their feedback was instrumental to the development of this article.

## Funding

This article is part of the Decade of Vaccine Economics (DOVE) project, funded under a multi-project grant by the 10.13039/100000865Bill & Melinda Gates Foundation, Seattle, WA (OPP112821). The project includes empirical assessments of the cost of pneumonia, diarrhea and measles in Uganda and Bangladesh, conducted by the International Vaccine Access Center at Johns Hopkins Bloomberg School of Public Health, Makerere University School of Public Health, and the International Diarrheal Disease Research Centre, Bangladesh.

## Declaration of Competing Interest

The authors declare the following financial interests/personal relationships which may be considered as potential competing interests: At the time the study was conducted, all co-authors received funds from the Bill & Melinda Gates Foundation. At the time of the development of this manuscript, Dagna Constenla was employee of GSK and holds stock option as an employee of GSK.
