# Supplementary material for: The economic burden of measles in children under five in Uganda
Source: Vaccine X. 2020 Sep 9;6:100077. doi: 10.1016/j.jvacx.2020.100077 (PMC7548439; doi:10.1016/j.jvacx.2020.100077)
Supplement: Supplementary data 1 [file mmc1.docx]

**Supplementary methods and figures**

***Healthcare facility sampling***

To adequately generate a national estimate of the cost of measles (and, in a separate analysis, pneumonia, and diarrhea), we selected one district per region for a total of four districts, and including districts where outbreaks of measles reported in the previous years. Specifically, we have obtained the reported caseload for each district by disease for 2011-2015 from the Ministry of Health District Health Information System II [1]. The selected districts included Gulu (Northern region), Jinja (Eastern), Mbarara (Western), and Wakiso (Central).

In each district, we selected 12 healthcare facilities representative of all three sectors (public, private for-profit, and private not-for-profit) and the different healthcare facility levels (primary, secondary, and tertiary). For the public sector, this meant selecting national or regional referral hospitals (tertiary), health centers IV (secondary), and health centers III and II (primary with and without inpatient capabilities, respectively). For the private sector, healthcare facilities with and without inpatient capabilities, equivalent to those in the public sector, were selected. We have selected districts and healthcare facilities where significant disease burden has been reported, noting that facility caseload reflects a mixture of factors such as the effectiveness of reporting, immunization coverage, living conditions, and population access to facilities.

By the end of the data collection period, 20 of the 48 healthcare facilities had reported measles cases. Cost data from those 20 facilities were included in the analysis and included in the calculation of societal costs, weighted by the number of cases reported in our sample. Eight of the 20 facilities were from the public sector; their data were used to calculate government costs. Below is a description of the types of health facility types.

| **Health Facility Types** | |
| --- | --- |
| **National referral hospital** | Serves the entire country; acts as a referral center for regional referral hospitals; offers a full range of services |
| **Regional referral hospital** | Serves a catchment populations of up to 2,000,000; acts as a referral center for district hospitals; offers a full range of inpatient and outpatient services, laboratory services, a subset of specialty services and higher-level surgical services than what is found at district hospitals |
| **Health center IV** | Serve as the highest non-hospital referral facilities at the sub-district level with a catchment population of around 100,000; offers basic preventive and curative outpatient and inpatient services, and second-level referral services (C-sections, blood transfusions, and other life-saving medical/surgical care) |
| **Health center III** | Serves a catchment population of up to 20,000; provides supervision of and referral services to health center II under their management; offers basic outpatient and inpatient services; some provide laboratory services |
| **Health center II** | Serve as basic health centers and interface to the formal health sector for populations of about 5,000; largely provides only outpatient services |
| **Pharmacies** | Privately owned, managed, and nationally registered; largely dispensing medications for a fee |

*Source: IHME ABCE Report: Assessing Facility Capacity, Costs of Care, and Patient Perspectives, Uganda* [2]

**Additional references**:

[1] District Health Information System (DHIS) II, Ministry of Health Uganda 2015.

[2] Health Service Provision in Uganda: Assessing Facility Capacity, Costs of Care, and Patient Perspectives, in: I.f.H.M.a.E. (IHME) (Ed.) IHME, Seattle, WA, 2014.

***Facility weights applied to generate societal costs***

The weigh applied to each public healthcare facility to overall societal costs is the number of caregivers interviewed at each facility:

| **District / Facility** | **Number of caregivers** |
| --- | --- |
| **Gulu district** | **0** |
|  |  |
| **Jinja district** | **55** |
| Jinja Hospital (referral hospital) | 53 |
| Walukuba (HC IV) | 2 |
|  |  |
| **Mbarara district** | **3** |
| Bwizibwera (HC IV) | 1 |
| Mbarara Hospital (referral hospital) | 2 |
|  |  |
| **Wakiso district** | **43** |
| Entebbe Hospital (referral hospital) | 26 |
| Kira (HC III) | 8 |
| Nansana (HC II) | 1 |
| Wakiso (HC IV) | 8 |

The size of the health facility and associated catchment contributed to the difference in caregivers interviewed.

***Table S1: Summary statistics for each asset quintile (2018 US dollars and UGX)***

| ***Median values*** | ***Wealth quintiles based on asset scores (principal component analysis)*** | | | | |
| --- | --- | --- | --- | --- | --- |
|  | *Poorest* |  |  |  | *Richest* |
|  | *1^st^* | *2^nd^* | *3^rd^* | *4^th^* | *5^th^* |
| **District** |  |  |  |  |  |
| Gulu | 1 | 0 | 0 | 0 | 0 |
| Jinja | 25 | 16 | 13 | 13 | 18 |
| Mbarara | 1 | 2 | 0 | 1 | 1 |
| Wakiso | 4 | 12 | 17 | 16 | 11 |
| **Residence** |  |  |  |  |  |
| Urban | 5 | 12 | 9 | 15 | 18 |
| Semi-urban | 0 | 0 | 5 | 6 | 7 |
| Rural | 26 | 18 | 16 | 9 | 5 |
| **Monthly income of:** |  |  |  |  |  |
| Head of the household | $48.30 | $100.62 | $87.20 | $134.16 | $160.99 |
|  | *UGX 180,000* | *UGX 375,000* | *UGX 325,000* | *UGX 500,000* | *UGX 600,000* |
| Caregiver | $0.00 | $0.00 | $16.10 | $28.17 | $21.46 |
|  | *UGX 0* | *UGX 0* | *UGX 60,000* | *UGX 105,000* | *UGX 80,000* |
| Household | $53.66 | $120.74 | $120.74 | $160.99 | $201.23 |
|  | *UGX 200,000* | *UGX 450,000* | *UGX 450,000* | *UGX 600,000* | *UGX 750,000* |
| **Monthly household expenditures** |  |  |  |  |  |
| All expenditures | $21.73 | $26.29 | $38.23 | $45.50 | $66.83 |
|  | *UGX 81,000* | *UGX 98,000* | *UGX 142,500* | *UGX 169,583* | *UGX 249,083* |
| Excluding food | $13.42 | $13.92 | $21.26 | $28.17 | $44.25 |
|  | *UGX 50,000* | *UGX 51,875* | *UGX 79,250* | *UGX 105,000* | *UGX 164,917* |

Notes: Income and expenditures (consumption) were recorded in Ugandan Shillings (UGX). The conversion rate used was the annual average of $1 = UGX 3,272 in 2018. The average monthly income of the head of the household was used to estimate indirect costs associated with the episode of pneumonia.

***Figure S1: Households financial coping mechanism related to an episode of measles by asset quintile***

***Table S2: Total household costs for an episode of measles (2018 UGX)***

|  |  |  | | | | | **INPATIENT CARE** | | | | |  | | | | |
| --- | --- | --- | --- | --- | --- | --- | --- | --- | --- | --- | --- | --- | --- | --- | --- | --- |
| **Timing** | **Cost** | **Public healthcare facilities (n=84)** | | | | | **Private for-profit healthcare facilities (n=6)** | | | | | **Private not-for-profit healthcare facilities (n=32)** | | | | |
|  |  | Mean | SE | 95% CI | | n(c>0) | Mean | SE | 95% CI | | n(c>0) | Mean | SE | 95% CI | | n(c>0) |
| **Before current visit^[A]^** | Direct medical | 4560 | 2130 | 323 | 8796 | 14 | 0 | 0 | 0 | 0 | 0 | 1953 | 888 | 142 | 3764 | 6 |
|  | Direct non-medical | 2476 | 706 | 1071 | 3881 | 21 | 10200 | 5507 | -3955 | 24355 | 3 | 3141 | 1498 | 86 | 6196 | 7 |
|  | Indirect | 3694 | 1192 | 1322 | 6067 | 30 | 5815 | 3453 | -3772 | 15401 | 3 | 11209 | 5934 | -909 | 23327 | 9 |
|  | *Time loss [days]* | 0.34 | 0.10 | 0.14 | 0.54 | 31 | 0.33 | 0.19 | -0.15 | 0.80 | 4 | 0.45 | 0.16 | 0.12 | 0.79 | 9 |
| **Current visit** | Direct medical | 14208 | 1881 | 10467 | 17949 | 57 | 47500 | 31695 | -33975 | 128975 | 4 | 50125 | 8620 | 32544 | 67706 | 26 |
|  | Direct non-medical | 29515 | 2122 | 25296 | 33735 | 84 | 62167 | 18264 | 15218 | 109115 | 6 | 26781 | 4857 | 16876 | 36687 | 31 |
|  | Indirect | 52685 | 8834 | 35106 | 70264 | 80 | 125833 | 44076 | 3458 | 248209 | 5 | 66283 | 17753 | 30026 | 102540 | 30 |
|  | *Time loss [days]* | 3.30 | 0.22 | 2.87 | 3.74 | 83 | 3.27 | 0.31 | 2.47 | 4.07 | 6 | 3.98 | 0.54 | 2.88 | 5.08 | 32 |
| **Follow-up^[A]^** | Direct medical | 8384 | 1783 | 4830 | 11938 | 28 | 9167 | 9167 | -14397 | 32730 | 1 | 34808 | 4645 | 25241 | 44374 | 18 |
|  | Direct non-medical | 26658 | 3217 | 20245 | 33070 | 48 | 11833 | 11833 | -18585 | 42252 | 1 | 20808 | 3128 | 14366 | 27249 | 21 |
|  | Indirect | 34697 | 6798 | 21139 | 48254 | 38 | 114333 | 114333 | -203107 | 431774 | 1 | 52127 | 9984 | 31521 | 72734 | 20 |
|  | *Time loss [days]* | 2.94 | 0.42 | 2.10 | 3.77 | 39 | 0.68 | 0.68 | -1.07 | 2.43 | 1 | 5.07 | 0.67 | 3.69 | 6.46 | 21 |
| **Total out-of-pocket expenses** | | **81212** | **5398** | **70476** | **91948** | **84** | **140867** | **31547** | **59772** | **221961** | **6** | **127188** | **10883** | **104992** | **149383** | **32** |
| **Total economic cost** | | **164905** | **13607** | **137842** | **191968** | **84** | **345851** | **157404** | **-58768** | **750470** | **6** | **242982** | **21391** | **199355** | **286610** | **32** |
|  |  |  | | | | | **OUTPATIENT CARE** | | | | |  | | | | |
| **Timing** | **Cost** | **Public healthcare facilities (n=17)** | | | | | **Private for-profit healthcare facilities (n=5)** | | | | | **Private not-for-profit healthcare facilities (n=7)** | | | | |
|  |  | Mean | SE | 95% CI | | n(c>0) | Mean | SE | 95% CI | | n(c>0) | Mean | SE | 95% CI | | n(c>0) |
| **Before current visit^[A]^** | Direct medical | 0 | 0 | 0 | 0 | 0 | 1400 | 1400 | -2487 | 5287 | 1 | 6429 | 6429 | -9302 | 22159 | **1** |
|  | Direct non-medical | 0 | 0 | 0 | 0 | 0 | 4800 | 4800 | -8527 | 18127 | 1 | 0 | 0 | 0 | 0 | **0** |
|  | Indirect | 17 | 17 | -20 | 55 | 1 | 185 | 185 | -404 | 775 | 1 | 119 | 119 | -172 | 410 | 1 |
|  | *Time loss [hours]* | 0.01 | 0.01 | -0.02 | 0.05 | 1 | 0.47 | 0.39 | -0.61 | 1.55 | 2 | 0.05 | 0.05 | -0.07 | 0.16 | 1 |
| **Current visit** | Direct medical | 1088 | 670 | -332 | 2509 | 3 | 61600 | 12560 | 26727 | 96473 | 5 | 8957 | 6231 | -6291 | 24205 | **2** |
|  | Direct non-medical | 6500 | 2488 | 1225 | 11775 | 14 | 7000 | 1817 | 1956 | 12044 | 5 | 1286 | 993 | -1144 | 3716 | **2** |
|  | Indirect | 7069 | 1194 | 4489 | 9649 | 14 | 8403 | 795 | 5873 | 10932 | 4 | 14705 | 6417 | -998 | 30408 | 7 |
|  | *Time loss [hours]* | 2.77 | 0.31 | 2.13 | 3.42 | 17 | 3.00 | 0.53 | 1.54 | 4.46 | 5 | 3.62 | 0.62 | 2.11 | 5.13 | 7 |
| **Follow-up^[A]^** | Direct medical | 1765 | 1765 | -1976 | 5506 | 1 | 62920 | 17904 | 13210 | 112631 | 4 | 0 | 0 | 0 | 0 | **0** |
|  | Direct non-medical | 118 | 118 | -132 | 367 | 1 | 6800 | 2223 | 629 | 12971 | 4 | 0 | 0 | 0 | 0 | **0** |
|  | Indirect | 104 | 104 | -121 | 329 | 1 | 8993 | 1257 | 4993 | 12993 | 4 | 0 | 0 | 0 | 0 | 0 |
|  | *Time loss [hours]* | 0.13 | 0.13 | -0.14 | 0.40 | 1 | 2.70 | 0.87 | 0.29 | 5.11 | 4 | 0.00 | 0.00 | 0.00 | 0.00 | 0 |
| **Total out-of-pocket expenses** | | **9471** | **2938** | **3242** | **15699** | **15** | **144520** | **27432** | **68356** | **220684** | **5** | **16671** | **10953** | **-10130** | **43473** | **2** |
| **Total economic cost** | | **15392** | **3631** | **7695** | **23089** | **17** | **158585** | **30563** | **73729** | **243441** | **5** | **31496** | **11773** | **2687** | **60304** | **7** |

Notes: SE, Standard Error; n, number of caregivers; n(c>0), number of caregivers who incurred costs greater than UGX 0.
^[A]^ Includes costs incurred at public and private healthcare facilities and providers.

***Table S3: Differences in household costs across caregiver characteristics (2018 UGX)***

| **Characteristic** | **n** | **Direct costs** | | | **Indirect costs** | | | **Total costs** | | |
| --- | --- | --- | --- | --- | --- | --- | --- | --- | --- | --- |
|  |  | Mean | SE | p-value^[B]^ | Mean | SE | p-value^[B]^ | Mean | SE | p-value^[B]^ |
| **Age group** |  |  |  |  |  |  |  |  |  |  |
| < 6 months | 13 | 57500 | 16782 | 0.015 | 41133 | 13569 | 0.195^[C]^ | 98633 | 27060 | 0.019^[C]^ |
| 6-11 months | 42 | 111354 | 10937 |  | 116613 | 27155 |  | 227967 | 32627 |  |
| 12-24 months | 49 | 76180 | 8518 |  | 69057 | 11472 |  | 145237 | 15977 |  |
| > 24 months | 47 | 76722 | 8784 |  | 85659 | 17177 |  | 162380 | 19205 |  |
| **Gender (child)** |  |  |  |  |  |  |  |  |  |  |
| Female | 70 | 84105 | 8232 | 0.454 | 78371 | 12330 | 0.617^[C]^ | 162475 | 14960 | 0.731^[C]^ |
| Male | 77 | 87397 | 7194 |  | 94573 | 16958 |  | 181971 | 21105 |  |
| **Gender (caregiver)** |  |  |  |  |  |  |  |  |  |  |
| Female | 142 | 83103 | 5317 | 0.256 | 88224 | 10986 | 0.835^[C]^ | 171327 | 13473 | 0.817 |
| Male | 9 | 120250 | 30883 |  | 52289 | 12074 |  | 172539 | 33925 |  |
| **Study area** |  |  |  |  |  |  |  |  |  |  |
| Gulu | 1 | 33000 | . | 0.000^[C]^ | 452083 | . | 0.000^[C]^ | 485083 | . | 0.000^[C]^ |
| Jinja | 85 | 98279 | 5963 |  | 99359 | 14233 |  | 197639 | 17186 |  |
| Mbarara | 5 | 91100 | 54445 |  | 112697 | 97590 |  | 203797 | 113396 |  |
| Wakiso | 60 | 65748 | 9057 |  | 56987 | 12331 |  | 122735 | 16639 |  |
| **Residence** |  |  |  |  |  |  |  |  |  |  |
| Rural | 74 | 96963 | 7571 | 0.080 | 83914 | 13609 | 0.904 | 180878 | 16857 | 0.828 |
| Semi-urban | 18 | 84533 | 18829 |  | 99740 | 39314 |  | 184273 | 42140 |  |
| Urban | 59 | 71802 | 7871 |  | 85391 | 17071 |  | 157193 | 21699 |  |
| **Type of visit** |  |  |  |  |  |  |  |  |  |  |
| Inpatient | 122 | 95393 | 5465 | 0.000 | 102270 | 12123 | 0.000^[C]^ | 197663 | 14200 | 0.000^[C]^ |
| Outpatient | 29 | 37472 | 12456 |  | 10991 | 2034 |  | 48463 | 13163 |  |
| **Facilities** |  |  |  |  |  |  |  |  |  |  |
| Public | 101 | 69540 | 5347 | 0.000 | 75062 | 10457 | 0.373^[C]^ | 144602 | 13174 | 0.002^[C]^ |
| PFP | 11 | 159756 | 20225 |  | 144470 | 90969 |  | 304226 | 102953 |  |
| PNFP | 39 | 106676 | 11686 |  | 100242 | 19716 |  | 206918 | 22492 |  |
| **Length of stay (IPD)^[A]^** |  |  |  |  |  |  |  |  |  |  |
| < 3 days | 103 | 79965 | 7283 | 0.019^[C]^ | 83037 | 20049 | 0.207 | 163002 | 24911 | 0.044^[C]^ |
| >= 3 days | 18 | 105069 | 7437 |  | 115000 | 15301 |  | 220068 | 16931 |  |
| **Asset quintiles** |  |  |  |  |  |  |  |  |  |  |
| Poorest | 31 | 111194 | 10732 | 0.028 | 75296 | 16896 | 0.958^[C]^ | 186489 | 19715 | 0.309^[C]^ |
| 2nd | 30 | 69886 | 12194 |  | 103898 | 28729 |  | 173784 | 34944 |  |
| 3rd | 30 | 62821 | 8817 |  | 85397 | 22176 |  | 148219 | 24895 |  |
| 4th | 30 | 84904 | 10736 |  | 70731 | 10802 |  | 155634 | 18812 |  |
| Richest | 30 | 94379 | 14750 |  | 96291 | 32606 |  | 190671 | 40798 |  |

Notes: SE, Standard Error; n, number of caregivers.
^[A]^ Length of stay includes only hospitalized cases of measles (n = 121 + 1 with missing length of stay).
^[B]^ Based on one-way ANOVA (age group, study area and asset quintile) and independent t-test for the others.
^[C]^ Based on Kruskal-Wallis rank test.

***Table S4: Government costs for an episode of measles (2018 UGX)***

| **Cost** | **Inpatient case** | | | | **Outpatient case** | | | |
| --- | --- | --- | --- | --- | --- | --- | --- | --- |
|  | *Public Healthcare Centre II* | | | | | | | |
|  | Mean | SE | 95% CI | | Mean | SE | 95% CI | |
|  | n = 0; hc = 0 | | | | n = 1; hc = 1 | | | |
| Capital | . | . | . | . | 0 | . | . | . |
| Overhead | . | . | . | . | 66 | . | . | . |
| Labor | . | . | . | . | 1199 | . | . | . |
| Medications | . | . | . | . | 863 | . | . | . |
| **Total cost** | **.** | **.** | **.** | **.** | **2128** | **.** | **.** | **.** |
|  |  |  |  |  |  |  |  |  |
|  | *Public Healthcare Centre III* | | | | | | | |
|  | n = 0; hc = 0 | | | | n = 8; hc = 1 | | | |
| Capital | . | . | . | . | 0 | . | . | . |
| Overhead | . | . | . | . | 418 | . | . | . |
| Labor | . | . | . | . | 13699 | . | . | . |
| Medications | . | . | . | . | 1442 | . | . | . |
| **Total cost** | **.** | **.** | **.** | **.** | **15559** | **.** | **.** | **.** |
|  |  |  |  |  |  |  |  |  |
|  | *Public Healthcare Centre IV* | | | | | | | |
|  | n = 4; hc = 3 | | | | n = 7; hc = 2 | | | |
| Capital | 214 | 123 | -179 | 606 | 147 | 24 | 87 | 206 |
| Overhead | 1087 | 148 | 617 | 1557 | 384 | 51 | 259 | 509 |
| Labor | 61039 | 9615 | 30439 | 91639 | 25435 | 1494 | 21780 | 29091 |
| Medications | 460 | 80 | 207 | 714 | 2843 | 2226 | -2604 | 8289 |
| **Total cost** | **62799** | **9649** | **32091** | **93508** | **28809** | **759** | **26952** | **30665** |
|  |  |  |  |  |  |  |  |  |
|  | *Public Regional Referral Hospital* | | | | | | | |
|  | n = 80; hc = 3 | | | | n = 1; hc = 1 | | | |
| Capital | 372 | 30 | 313 | 431 | 0 | . | . | . |
| Overhead | 9236 | 261 | 8716 | 9755 | 2047 | . | . | . |
| Labor | 28925 | 1687 | 25566 | 32284 | 2040 | . | . | . |
| Medications | 6207 | 204 | 5801 | 6614 | 58 | . | . | . |
| **Total cost** | **44740** | **1722** | **41312** | **48167** | **4145** | **.** | **.** | **.** |

Note: **n** represents the number of caregivers interviewed at the facility level
**hc** represents the number of healthcare facilities included at the facility level

***Table S5: Household costs for a hospitalized episode of measles (2018 UGX), public and private for-profit healthcare facilities.***

| **Timing** | **Type** | **Cost** | **Public healthcare facilities (n=84)** | | | | | **Private for-profit healthcare facilities (n=6)** | | | | |
| --- | --- | --- | --- | --- | --- | --- | --- | --- | --- | --- | --- | --- |
|  |  |  | Mean | SE | 95% CI | | n(c>0) | Mean | SE | 95% CI | | n(c>0) |
| **Before current visit^A^** | **Direct medical costs** | Consultation | 536 | 397 | -255 | 1326 | 3 | 0 | 0 | 0 | 0 | 0 |
|  |  | Investigations | 0 | 0 | 0 | 0 | 0 | 0 | 0 | 0 | 0 | 0 |
|  |  | Medications | 4024 | 2105 | -163 | 8210 | 11 | 0 | 0 | 0 | 0 | 0 |
|  |  | Hospitalization | 0 | 0 | 0 | 0 | 0 | 0 | 0 | 0 | 0 | 0 |
|  |  | **Total** | **4560** | **2130** | **323** | **8796** | **14** | **0** | **0** | **0** | **0** | **0** |
|  | **Direct non-medical costs** | Transportation | 964 | 242 | 483 | 1446 | 21 | 2667 | 1333 | -761 | 6094 | 3 |
|  |  | Meals | 774 | 394 | -10 | 1557 | 7 | 7533 | 4743 | -4660 | 19727 | 3 |
|  |  | Other | 738 | 284 | 173 | 1303 | 9 | 0 | 0 | 0 | 0 | 0 |
|  |  | **Total** | **2476** | **706** | **1071** | **3881** | **21** | **10200** | **5507** | **-3955** | **24355** | **3** |
|  | **Indirect costs** | Time loss [days] | 0.34 | 0.10 | 0.14 | 0.54 | 31 | 0.33 | 0.19 | -0.15 | 0.80 | 4 |
|  |  | Productivity loss | **3694** | **1192** | **1322** | **6067** | **30** | **5815** | **3453** | **-3772** | **15401** | **3** |
| **Current** | **Direct medical costs** | Consultation | 0 | 0 | 0 | 0 | 0 | 3333 | 2472 | -3021 | 9688 | 2 |
|  |  | Investigations | 310 | 242 | -173 | 792 | 3 | 4167 | 4167 | -6544 | 14877 | 1 |
|  |  | Medications | 13899 | 1865 | 10190 | 17608 | 57 | 40000 | 32660 | -43955 | 123955 | 2 |
|  |  | Hospitalization | 0 | 0 | 0 | 0 | 0 | 0 | 0 | 0 | 0 | 0 |
|  |  | **Total** | **14208** | **1881** | **10467** | **17949** | **57** | **47500** | **31695** | **-33975** | **128975** | **4** |
|  | **Direct non-medical costs** | Transportation | 6042 | 774 | 4502 | 7581 | 81 | 9667 | 2155 | 4127 | 15207 | 6 |
|  |  | Meals | 18530 | 1572 | 15402 | 21657 | 79 | 41667 | 21003 | -12322 | 95656 | 3 |
|  |  | Other | 4944 | 608 | 3734 | 6154 | 62 | 10833 | 10833 | -17015 | 38681 | 1 |
|  |  | **Total** | **29515** | **2122** | **25296** | **33735** | **84** | **62167** | **18264** | **15218** | **109115** | **6** |
|  | **Indirect costs** | Time loss [days] | 3.30 | 0.22 | 2.87 | 3.74 | 83 | 3.27 | 0.31 | 2.47 | 4.07 | 6 |
|  |  | Productivity loss | **52685** | **8834** | **35106** | **70264** | **80** | **125833** | **44076** | **3458** | **248209** | **5** |
| **Follow-up^A^** | **Direct medical costs** | Consultation | 0 | 0 | 0 | 0 | 0 | 2500 | 2500 | -3926 | 8926 | 1 |
|  |  | Investigations | 0 | 0 | 0 | 0 | 0 | 0 | 0 | 0 | 0 | 0 |
|  |  | Medications | 8384 | 1783 | 4830 | 11938 | 28 | 6667 | 6667 | -10471 | 23804 | 1 |
|  |  | Hospitalization | 0 | 0 | 0 | 0 | 0 | 0 | 0 | 0 | 0 | 0 |
|  |  | **Total** | **8384** | **1783** | **4830** | **11938** | **28** | **9167** | **9167** | **-14397** | **32730** | **1** |
|  | **Direct non-medical costs** | Transportation | 2527 | 538 | 1454 | 3600 | 36 | 1000 | 1000 | -1571 | 3571 | 1 |
|  |  | Meals | 17610 | 2372 | 12882 | 22337 | 44 | 0 | 0 | 0 | 0 | 0 |
|  |  | Other | 6521 | 1023 | 4481 | 8560 | 40 | 10833 | 10833 | -17015 | 38681 | 1 |
|  |  | **Total** | **26658** | **3217** | **20245** | **33070** | **48** | **11833** | **11833** | **-18585** | **42252** | **1** |
|  | **Indirect costs** | Time loss [days] | 2.94 | 0.42 | 2.10 | 3.77 | 39 | 0.68 | 0.68 | -1.07 | 2.43 | 1 |
|  |  | Productivity loss | **34697** | **6798** | **21139** | **48254** | **38** | **114333** | **114333** | **-203107** | **431774** | **1** |
| **Total direct (financial) cost** | | | **81212** | **5398** | **70476** | **91948** | **84** | **140867** | **31547** | **59772** | **221961** | **6** |
| **Total economic cost** | | | **164905** | **13607** | **137842** | **191968** | **84** | **345851** | **157404** | **-58768** | **750470** | **6** |

Notes: SE, Standard Error; n, number of caregivers; n(c>0), number of caregivers who incurred costs greater than UGX 0.
^A^ Includes costs incurred at public and private healthcare facilities and providers.

***Table S6: Household costs for a hospitalized episode of measles (2018 UGX), private not-for-profit healthcare facilities.***

| **Timing** | **Type** | **Cost** | **Private not-for-profit healthcare facilities (n=32)** | | | | |
| --- | --- | --- | --- | --- | --- | --- | --- |
|  |  |  | Mean | SE | 95% CI | | n(c>0) |
| **Before current visit^A^** | **Direct medical costs** | Consultation | 625 | 625 | -650 | 1900 | 1 |
|  |  | Investigations | 94 | 94 | -97 | 285 | 1 |
|  |  | Medications | 1234 | 671 | -134 | 2603 | 4 |
|  |  | Hospitalization | 0 | 0 | 0 | 0 | 0 |
|  |  | **Total** | **1953** | **888** | **142** | **3764** | **6** |
|  | **Direct non-medical costs** | Transportation | 1391 | 976 | -600 | 3382 | 5 |
|  |  | Meals | 813 | 540 | -288 | 1913 | 3 |
|  |  | Other | 938 | 475 | -31 | 1906 | 4 |
|  |  | **Total** | **3141** | **1498** | **86** | **6196** | **7** |
|  | **Indirect costs** | Time loss [days] | 0.45 | 0.16 | 0.12 | 0.79 | 9 |
|  |  | Productivity loss | **11209** | **5934** | **-909** | **23327** | **9** |
| **Current** | **Direct medical costs** | Consultation | 31406 | 4154 | 22934 | 39879 | 23 |
|  |  | Investigations | 4000 | 2303 | -697 | 8697 | 5 |
|  |  | Medications | 10500 | 7179 | -4142 | 25142 | 3 |
|  |  | Hospitalization | 4219 | 4060 | -4063 | 12500 | 2 |
|  |  | **Total** | **50125** | **8620** | **32544** | **67706** | **26** |
|  | **Direct non-medical costs** | Transportation | 9250 | 2246 | 4668 | 13832 | 29 |
|  |  | Meals | 7125 | 2526 | 1972 | 12278 | 8 |
|  |  | Other | 10406 | 2102 | 6119 | 14694 | 21 |
|  |  | **Total** | **26781** | **4857** | **16876** | **36687** | **31** |
|  | **Indirect costs** | Time loss [days] | 3.98 | 0.54 | 2.88 | 5.08 | 32 |
|  |  | Productivity loss | **66283** | **17753** | **30026** | **102540** | **30** |
| **Follow-up^A^** | **Direct medical costs** | Consultation | 34808 | 4645 | 25241 | 44374 | 18 |
|  |  | Investigations | 0 | 0 | 0 | 0 | 0 |
|  |  | Medications | 0 | 0 | 0 | 0 | 0 |
|  |  | Hospitalization | 0 | 0 | 0 | 0 | 0 |
|  |  | **Total** | **34808** | **4645** | **25241** | **44374** | **18** |
|  | **Direct non-medical costs** | Transportation | 5423 | 1045 | 3270 | 7576 | 20 |
|  |  | Meals | 1731 | 1207 | -755 | 4217 | 2 |
|  |  | Other | 13654 | 2550 | 8401 | 18906 | 18 |
|  |  | **Total** | **20808** | **3128** | **14366** | **27249** | **21** |
|  | **Indirect costs** | Time loss [days] | 5.07 | 0.67 | 3.69 | 6.46 | 21 |
|  |  | Productivity loss | **52127** | **9984** | **31521** | **72734** | **20** |
| **Total direct (financial) cost** | | | **127188** | **10883** | **104992** | **149383** | **32** |
| **Total economic cost** | | | **242982** | **21391** | **199355** | **286610** | **32** |

Notes: SE, Standard Error; n, number of caregivers; n(c>0), number of caregivers who incurred costs greater than UGX 0.
^A^ Includes costs incurred at public and private healthcare facilities and providers.

***Table S7: Household costs for an outpatient episode of measles (2018 UGX), public and private for-profit healthcare facilities.***

| **Timing** | **Type** | **Cost** | **Public healthcare facilities (n=17)** | | | | | **Private for-profit healthcare facilities (n=5)** | | | | |
| --- | --- | --- | --- | --- | --- | --- | --- | --- | --- | --- | --- | --- |
|  |  |  | Mean | SE | 95% CI | | n(c>0) | Mean | SE | 95% CI | | n(c>0) |
| **Before current visit^A^** | **Direct medical costs** | Consultation | 0 | 0 | 0 | 0 | 0 | 0 | 0 | 0 | 0 | 0 |
|  |  | Investigations | 0 | 0 | 0 | 0 | 0 | 0 | 0 | 0 | 0 | 0 |
|  |  | Medications | 0 | 0 | 0 | 0 | 0 | 1400 | 1400 | -2487 | 5287 | 1 |
|  |  | **Total** | **0** | **0** | **0** | **0** | **0** | **1400** | **1400** | **-2487** | **5287** | **1** |
|  | **Direct non-medical costs** | Transportation | 0 | 0 | 0 | 0 | 0 | 800 | 800 | -1421 | 3021 | 1 |
|  |  | Meals | 0 | 0 | 0 | 0 | 0 | 4000 | 4000 | -7106 | 15106 | 1 |
|  |  | Other | 0 | 0 | 0 | 0 | 0 | 0 | 0 | 0 | 0 | 0 |
|  |  | **Total** | **0** | **0** | **0** | **0** | **0** | **4800** | **4800** | **-8527** | **18127** | **1** |
|  | **Indirect costs** | Time loss [hours] | 0.01 | 0.01 | -0.02 | 0.05 | 1 | 0.47 | 0.39 | -0.61 | 1.55 | 2 |
|  |  | Productivity loss | **17** | **17** | **-20** | **55** | **1** | **185** | **185** | **-404** | **775** | **1** |
| **Current** | **Direct medical costs** | Consultation | 0 | 0 | 0 | 0 | 0 | 13600 | 1400 | 9713 | 17487 | 5 |
|  |  | Investigations | 0 | 0 | 0 | 0 | 0 | 11000 | 2915 | 2905 | 19095 | 4 |
|  |  | Medications | 1088 | 670 | -332 | 2509 | 3 | 37000 | 10826 | 6942 | 67058 | 4 |
|  |  | **Total** | **1088** | **670** | **-332** | **2509** | **3** | **61600** | **12560** | **26727** | **96473** | **5** |
|  | **Direct non-medical costs** | Transportation | 1971 | 328 | 1276 | 2666 | 14 | 5400 | 1249 | 1932 | 8868 | 5 |
|  |  | Meals | 529 | 273 | -49 | 1108 | 4 | 1600 | 1030 | -1259 | 4459 | 2 |
|  |  | Other | 4000 | 2292 | -859 | 8859 | 11 | 0 | 0 | 0 | 0 | 0 |
|  |  | **Total** | **6500** | **2488** | **1225** | **11775** | **14** | **7000** | **1817** | **1956** | **12044** | **5** |
|  | **Indirect costs** | Time loss [hours] | 2.77 | 0.31 | 2.13 | 3.42 | 17 | 3.00 | 0.53 | 1.54 | 4.46 | 5 |
|  |  | Productivity loss | **7069** | **1194** | **4489** | **9649** | **14** | **8403** | **795** | **5873** | **10932** | **4** |
| **Follow-up^A^** | **Direct medical costs** | Consultation | 0 | 0 | 0 | 0 | 0 | 12000 | 3000 | 3671 | 20329 | 4 |
|  |  | Investigations | 0 | 0 | 0 | 0 | 0 | 9000 | 3674 | -1201 | 19201 | 3 |
|  |  | Medications | 1765 | 1765 | -1976 | 5506 | 1 | 41920 | 12901 | 6102 | 77738 | 4 |
|  |  | **Total** | **1765** | **1765** | **-1976** | **5506** | **1** | **62920** | **17904** | **13210** | **112631** | **4** |
|  | **Direct non-medical costs** | Transportation | 59 | 59 | -66 | 184 | 1 | 5200 | 1744 | 359 | 10041 | 4 |
|  |  | Meals | 0 | 0 | 0 | 0 | 0 | 1600 | 1030 | -1259 | 4459 | 2 |
|  |  | Other | 59 | 59 | -66 | 184 | 1 | 0 | 0 | 0 | 0 | 0 |
|  |  | **Total** | **118** | **118** | **-132** | **367** | **1** | **6800** | **2223** | **629** | **12971** | **4** |
|  | **Indirect costs** | Time loss [hours] | 0.13 | 0.13 | -0.14 | 0.40 | 1 | 2.70 | 0.87 | 0.29 | 5.11 | 4 |
|  |  | Productivity loss | **104** | **104** | **-121** | **329** | **1** | **8993** | **1257** | **4993** | **12993** | **4** |
| **Total direct (financial) cost** | | | **9471** | **2938** | **3242** | **15699** | **15** | **144520** | **27432** | **68356** | **220684** | **5** |
| **Total economic cost** | | | **15392** | **3631** | **7695** | **23089** | **17** | **158585** | **30563** | **73729** | **243441** | **5** |

Notes: SE, Standard Error; n, number of caregivers; n(c>0), number of caregivers who incurred costs greater than UGX 0.
^A^ Includes costs incurred at public and private healthcare facilities and providers.

***Table S8: Household costs for an outpatient episode of measles (2018 UGX), private not-for-profit healthcare facilities.***

| **Timing** | **Type** | **Cost** | **Private not-for-profit healthcare facilities (n=7)** | | | | |
| --- | --- | --- | --- | --- | --- | --- | --- |
|  |  |  | Mean | SE | 95% CI | | n(c>0) |
| **Before current visit^A^** | **Direct medical costs** | Consultation | 0 | 0 | 0 | 0 | 0 |
|  |  | Investigations | 0 | 0 | 0 | 0 | 0 |
|  |  | Medications | 6429 | 6429 | -9302 | 22159 | 1 |
|  |  | **Total** | **6429** | **6429** | **-9302** | **22159** | **1** |
|  | **Direct non-medical costs** | Transportation | 0 | 0 | 0 | 0 | 0 |
|  |  | Meals | 0 | 0 | 0 | 0 | 0 |
|  |  | Other | 0 | 0 | 0 | 0 | 0 |
|  |  | **Total** | **0** | **0** | **0** | **0** | **0** |
|  | **Indirect costs** | Time loss [hours] | 0.05 | 0.05 | -0.07 | 0.16 | 1 |
|  |  | Productivity loss | **119** | **119** | **-172** | **410** | **1** |
| **Current** | **Direct medical costs** | Consultation | 714 | 714 | -1034 | 2462 | 1 |
|  |  | Investigations | 1714 | 1714 | -2480 | 5909 | 1 |
|  |  | Medications | 6529 | 4494 | -4467 | 17524 | 2 |
|  |  | **Total** | **8957** | **6231** | **-6291** | **24205** | **2** |
|  | **Direct non-medical costs** | Transportation | 286 | 286 | -413 | 985 | 1 |
|  |  | Meals | 286 | 286 | -413 | 985 | 1 |
|  |  | Other | 714 | 714 | -1034 | 2462 | 1 |
|  |  | **Total** | **1286** | **993** | **-1144** | **3716** | **2** |
|  | **Indirect costs** | Time loss [hours] | 3.62 | 0.62 | 2.11 | 5.13 | 7 |
|  |  | Productivity loss | **14705** | **6417** | **-998** | **30408** | **7** |
| **Follow-up^A^** | **Direct medical costs** | Consultation | 0 | 0 | 0 | 0 | 0 |
|  |  | Investigations | 0 | 0 | 0 | 0 | 0 |
|  |  | Medications | 0 | 0 | 0 | 0 | 0 |
|  |  | **Total** | **0** | **0** | **0** | **0** | **0** |
|  | **Direct non-medical costs** | Transportation | 0 | 0 | 0 | 0 | 0 |
|  |  | Meals | 0 | 0 | 0 | 0 | 0 |
|  |  | Other | 0 | 0 | 0 | 0 | 0 |
|  |  | **Total** | **0** | **0** | **0** | **0** | **0** |
|  | **Indirect costs** | Time loss [hours] | 0.00 | 0.00 | 0.00 | 0.00 | 0 |
|  |  | Productivity loss | **0** | **0** | **0** | **0** | **0** |
| **Total direct (financial) cost** | | | **16671** | **10953** | **-10130** | **43473** | **2** |
| **Total economic cost** | | | **31496** | **11773** | **2687** | **60304** | **7** |

Notes: SE, Standard Error; n, number of caregivers; n(c>0), number of caregivers who incurred costs greater than UGX 0.
^A^ Includes costs incurred at public and private healthcare facilities and providers.
